# Pulmonary Surfactant and Drug Delivery: An Interface-Assisted Carrier to Deliver Surfactant Protein SP-D Into the Airways

**DOI:** 10.3389/fbioe.2020.613276

**Published:** 2021-01-18

**Authors:** Cristina García-Mouton, Alberto Hidalgo, Raquel Arroyo, Mercedes Echaide, Antonio Cruz, Jesús Pérez-Gil

**Affiliations:** Department of Biochemistry and Molecular Biology, Faculty of Biology, Research Institute “Hospital 12 de Octubre (imas12),” Complutense University, Madrid, Spain

**Keywords:** pulmonary surfactant, interfacial delivery, respiratory drug delivery, air-liquid interface, lipid-protein interaction

## Abstract

This work is focused on the potential use of pulmonary surfactant to deliver full-length recombinant human surfactant protein SP-D (rhSP-D) using the respiratory air-liquid interface as a shuttle. Surfactant protein D (SP-D) is a collectin protein present in the pulmonary surfactant (PS) system, involved in innate immune defense and surfactant homeostasis. It has been recently suggested as a potential therapeutic to alleviate inflammatory responses and lung diseases in preterm infants suffering from respiratory distress syndrome (RDS) or bronchopulmonary dysplasia (BPD). However, none of the current clinical surfactants used for surfactant replacement therapy (SRT) to treat RDS contain SP-D. The interaction of SP-D with surfactant components, the potential of PS as a respiratory drug delivery system and the possibility to produce recombinant versions of human SP-D, brings the possibility of delivering clinical surfactants supplemented with SP-D. Here, we used an *in vitro* setup that somehow emulates the respiratory air-liquid interface to explore this novel approach. It consists in two different compartments connected with a hydrated paper bridge forming a continuous interface. We firstly analyzed the adsorption and spreading of rhSP-D alone from one compartment to another over the air-liquid interface, observing low interfacial activity. Then, we studied the interfacial spreading of the protein co-administered with PS, both at different time periods or as a mixed formulation, and which oligomeric forms of rhSP-D better traveled associated with PS. The results presented here demonstrated that PS may transport rhSP-D long distances over air-liquid interfaces, either as a mixed formulation or separately in a close window time, opening the doors to empower the current clinical surfactants and SRT.

## Introduction

Surfactant protein D (SP-D) is a C-type calcium-dependent lectin that belongs to the collectin family. It is involved in the innate immune properties of pulmonary surfactant (PS) (Crouch et al., 1994; Crouch, 2000) and contributes to alveolar and surfactant homeostasis (Korfhagen et al., 1998). PS is a lipid-protein material essential for the process of breathing that has been proposed as potent drug delivery system (Van't Veen et al., 1996; De Backer et al., 2013; Banaschewski et al., 2015; Hidalgo et al., 2015, 2017). PS is mainly composed by lipids (90% by mass), mainly phospholipids, and four different proteins (6–8% by mass): two hydrophobic (SP-B and SP-C) and two hydrophilic (SP-A and SP-D) (Pérez-Gil, 2008; Parra and Pérez-Gil, 2015). SP-B and SP-C are essential for the maintenance and organization of PS at the air-liquid interface, while SP-A and SP-D are mostly involved in innate immune defense (Perez-Gil and Weaver, 2010). PS enables the process of breathing by lowering the surface tension of the layer of water covering the whole respiratory surface, minimizing the work of breathing and avoiding the alveolar collapse. Apart from the interfacial and immune defense functions, its composition and interfacial properties confers PS the possibility to spread efficiently over air-liquid interfaces and transport therapeutic molecules by surfing the respiratory surface, what has been called an interfacial delivery (Hidalgo et al., 2020).

As the rest of collectins, SP-D monomers (43 kDa) contain four different structural domains: a short N-terminal region enriched in cysteines, a collagen-like domain of Gly-X-Y repetitions, a neck region with α-helical structure and a C-terminal carbohydrate recognition domain (CRD), which constitutes the key structure for most of the protein functions and interactions (Orgeig et al., 2011; Casals et al., 2018). Monomers may associate into trimers (130 kDa), constituting the minimal functional unit to allow the recognition of specific molecules through the CRD. Trimers can also associate forming hexamers, dodecamers (520 kDa) and the so-called “fuzzy balls,” which have been recently considered as the most potent oligomeric form of SP-D in bacterial aggregation (Arroyo et al., 2018, 2020). By recognizing a wide range of pathogens and foreign particles mostly through the CRD, SP-D promotes opsonization and aggregation and further clearance by phagocytic alveolar cells (Orgeig et al., 2011; Watson et al., 2019). Additionally, it also modulates the release of pro- and anti-inflammatory mediators via toll-like receptors and calreticulin/CD91 (Kingma and Whitsett, 2006; Sorensen, 2018).

Due to the immuno-modulatory and anti-inflammatory potential of SP-D, its delivery through the airways has been proposed in recent years as a potential therapeutic approach to alleviate inflammatory processes in the lungs. Since Clark and Reid highlighted in 2003 the potential benefits of delivering recombinant fragments of human SP-D (rfhSP-D) as a potential therapy to reduce inflammation in neonatal chronic lung disease, cystic fibrosis and emphysema (Clark and Reid, 2003), few works have explored this anti-inflammatory strategy and how this protein can be delivered through the airways. The instillation of rfhSP-D alone showed a reduction of inflammation derived from allergy in mice (Strong et al., 2003; Liu et al., 2005) or LPS in lambs (Ikegami et al., 2006) using recombinant fragments or full length recombinant human SP-D (rhSP-D), respectively. A recent study demonstrated the benefits of encapsulating SP-D in PLGA nanoparticles as a sustained release approach during several days from the administration (Cohen et al., 2020). In spite of the therapeutic effects of SP-D, the current commercially available clinical surfactants are all still lacking SP-D. However, since SP-D interacts with pulmonary surfactant components (Korfhagen et al., 1998), preferentially with phosphatidylinositol (PI) (Ogasawara et al., 1992), the possibility of delivering clinical surfactants supplemented with rhSP-D has also been explored showing enhanced anti-inflammatory effects of PS/SP-D formulations on ventilation- (Sato et al., 2010) and LPS-derived (Ikegami et al., 2007) inflammation in lambs and mice, respectively. However, to the best of our knowledge, the capability of PS to transport SP-D interfacially to optimize PS/SP-D formulations and delivery has not been studied.

Therefore, in the present study we have evaluated for the first time (1) the possibility of SP-D to adsorb into and spread over air-liquid interfaces, (2) whether PS enhances this process, and (3) the influence of PS structures to interact with the different oligomeric forms of SP-D (i.e., trimers, hexamers and “fuzzy balls”). To do so, we used self-designed vehiculization setups consisting in two aqueous compartments connected by an interfacial bridge, and different PS/SP-D preparations and modes of administration were tested.

## Materials and Methods

Unless otherwise indicated, all chemicals and reagents were purchased from commercial suppliers (i.e., Sigma-Aldrich®, Merck KGaA or Macron Fine Chemicals™). Water was filtered and treated with a Merck-Millipore Direct-Q3 purification system and further distilled for the surface balance experiments.

### Pulmonary Surfactant Preparations

#### Native Surfactant

Native pulmonary surfactant (NS) was isolated from bronchoalveolar lavage (BAL) of fresh slaughtered porcine lungs as previously described (Taeusch et al., 2005). Briefly, BAL was centrifuged at 1,000 g for 5 min to eliminate cells and tissue debris. Then, it was subsequently ultracentrifuged for 1 h at 100,000 g and 4°C. Pellets, containing the surfactant complexes, were resuspended in 16% NaBr 0.9% NaCl to perform a discontinuous NaBr density gradient at 120,000 g for 2 h at 4°C to purify the surfactant complexes from other cell membranes. After the gradient, pulmonary surfactant complexes, concentrated between the lighter (0.9% NaCl) and the medium dense solution (13% NaBr 0.9% NaCl), were homogenized with 0.9% NaCl and stored at −80°C until used.

#### Surfactant Organic Extract

Surfactant organic extract (OE), containing all the lipids plus the hydrophobic proteins SP-B and SP-C, was obtained following the organic extraction protocol established by Blight and Dyer (Bligh and Dyer, 1959). A mixture of chloroform/methanol/water (1:2:1 v/v/v) was added to the NS and incubated during 30 min at 37°C to allow protein flocculation. An additional volume of water and chloroform were added to the mixture and centrifuged 5 min at 3,000 g and 4°C. The fraction at the bottom containing the hydrophobic components of NS (organic phase) was collected. The upper fraction (aqueous phase) was subjected to two successive lavages by adding two volumes of chloroform and centrifuged 5 min at 3,000 g and 4°C. Finally, the material recovered was stored at −20°C until used.

To prepare aqueous suspensions from OE, proper amounts of the material were dried under a nitrogen stream and further vacuum for 2 h to form a dry film without organic solvent traces. The dried films were reconstituted by hydration with a buffer solution (5 mM Tris, 150 mM NaCl, pH 7.4) during 1 h at 45°C, shacking vigorously every 10 min. When needed, the aqueous solution was sonicated in ice during 2 min (burst for 0.6, and 0.4 s between bursts) at 65% amplitude for 7 cycles in a UP 200S sonifier, with a 2 mm microtip.

#### Poractant α

Poractant α, commercially available as Curosurf®, was obtained from Chiesi Farmaceutici S.p.A. (Parma, Italy) at a concentration of 80 mg/mL.

### Recombinant Human SP-D (rhSP-D)

rhSP-D was provided by Airway Therapeutics Inc. It has been produced and purified as previously described by Arroyo et al. (2018). All the different clones used have been previously analyzed by atomic force microscopy (AFM) to qualitatively and quantitatively characterize the oligomeric forms of the protein.

#### Fluorescent Labeling of rhSP-D

rhSP-D was conjugated with the amine-reactive fluorescent dye Alexa Fluor 488. First, the protein was exchanged to Hepes buffer (10 mM Hepes, 200 mM NaCl, 1 mM EDTA, pH 7.4) by dialysis at 4°C, in which the labeling reaction would take place. The proper amount of the probe, dissolved in methanol, to get a 1:20 (mol/mol) protein/probe ratio was dried under a nitrogen stream and under vacuum for 30 min and further dissolved in water. To shift the pH to values near 9 and activate amines, NaHCO_3_ was added to the solution. Then, the labeling reaction was performed for 1 h at room temperature with continuous stirring. Finally, to separate fluorescently labeled protein (F-rhSP-D) from the free probe, the solution was dialyzed against Histidine buffer (5 mM His, 200 mM NaCl, 1 mM EDTA, pH 6).

### Interfacial Assays

In the present study, the adsorption and spreading properties of rhSP-D by itself and the delivery capabilities of PS were characterized in Wilhelmy and vehiculization troughs.

#### Adsorption Tests

To evaluate the interfacial adsorption of the protein, experiments were performed using a single Wilhelmy trough (NIMA technologies, Coventry, UK). To do so, 1.8 mL of a buffered solution (5 mM Tris, 150 mM NaCl, pH 7.4) was placed in the Wilhelmy trough and an aqueous aliquot of 10 μL at 0.34 mg/mL (3.4 μg) of rhSP-D was injected into the subphase close to the surface, before monitoring the changes in surface pressure during 100 min with a pressure sensor (NIMA technologies, Coventry, UK). The subphase was constantly stirred to reduce diffusion limitation and thermostated at 25 ± 1°C.

#### Spreading and Vehiculization Assays

In order to explore the interfacial spreading capabilities of SP-D and its potential interface-assisted vehiculization by PS, an *in vitro* vehiculization setup was used (Yu and Possmayer, 2003; Hidalgo et al., 2017). Briefly, it consists in two different troughs containing a buffered solution (5 mM Tris, 150 mM NaCl, pH 7.4) connected by an interfacial bridge. One of the troughs is used as a donor (surface area: 315 mm^2^; subphase volume: 1.8 mL), somehow mimicking delivery at the upper airways, and the other as the recipient, which emulates the target surfaces at the distal airways and may vary depending on the experiment. Both troughs are connected by an interfacial bridge (6 cm length × 1 cm width), made of a hydrated filter paper (No. 1 Whatman filter paper), which creates a continuous air-liquid interface between both compartments, somehow recreating the conductive airways (Supplementary Figure 1). The filter paper was hydrated by submersion into the same buffer solution during 5 min before connecting both compartments. The samples were added directly onto the donor interface by drop deposition. This should simulate the arrival of surfactant or surfactant/drug drops, either upon nebulization or direct bolus deposition, into the upper airways. Changes in surface pressure were simultaneously monitored in both donor and recipient compartments (pressure sensors from NIMA technologies, Coventry, UK). An increase of surface pressure at the donor trough indicates that the sample adsorbs into the air-liquid interface. The increase of surface pressure at the recipient trough is a signal indicating that the sample can interfacially spread over the air-liquid interface. This interfacial spreading of material is likely governed by Marangoni convection. The surface tension gradient between both connected compartments leads to the spread of material from the donor, where the surface tension is lower (higher surface pressure) and near the equilibrium, to the recipient trough, where the surface tension is initially high (low surface pressure) (Borgas and Grotberg, 1988; Grotberg and Gaver III, 1996; Halpern et al., 1998). To determine whether PS can transport SP-D, the recipient interface was measured by fluorescence or visualized under transmission electron microscopy (TEM) or atomic force microscopy (AFM) as detailed in the next sections. The experimental temperature was maintained constant at 25 ± 1°C. Different vehiculization setups and samples were used for each assay:

##### Spreading Properties of rhSP-D Alone

An aliquot of 20 μL at 0.6 mg/mL (12 μg) of rhSP-D, an amount enough to have an excess of protein, was added by drop deposition onto the donor interface connected to a recipient trough with a surface area of 25 cm^2^ and 25 mL subphase volume. To determine the fluorescence of F-shSP-D, a smaller version of the recipient trough (surface area: 315 mm^2^; subphase volume: 1.8 mL) was used in order to collect the whole volume.

##### Interfacial Vehiculization of a Combined PS/rhSP-D Formulation

An aqueous suspension of OE was incubated with rhSP-D (1% by mass with respect to lipids) at 37°C for 30 min. In these experiments, a rhSP-D clone enriched with higher amounts of fuzzy ball oligomers (82% by weight of total protein mass) compared with the average quantity [29% weight (Arroyo et al., 2018)] was used to facilitate its detection and recognition under the microscopes. Then, an aqueous aliquot of 15 μL at 50 mg/mL (750 μg) of OE was added by drop deposition onto the donor interface connected to the recipient trough (surface area: 25 cm^2^; subphase volume: 25 mL). To visualize the protein under TEM and AFM, the interfacial films were transferred to carbon-coated cupper grids and mica plates, respectively, as explained in the next sections. In addition, to evaluate the differential vehiculization of the oligomeric forms of rhSP-D, the aqueous suspension of OE was sonicated or not prior incubation with 1% rhSP-D by mass. In this case, a Langmuir-Blodgett trough (surface area: 60–184 cm^2^; subphase volume: 350 mL) was used as recipient to transfer the interfacial film onto mica plates.

##### Co-administration of PS and rhSP-D

In an attempt to strategize a sequential co-administration of PS and rhSP-D and understand the mechanisms of the interaction and interfacial spreading of PS and rhSP-D, we added both materials sequentially instead of as a combined formulation to the donor compartment connected to the recipient trough (surface area: 25 cm^2^; subphase volume: 25 mL). The clinical surfactant Curosurf (50 μL at 80 mg/mL; 4 mg) and the fluorescent derivative of rhSP-D (15 μL at 1 mg/mL; 15 μg) were used for these experiments. In a first scenario, Curosurf was firstly added by drop deposition onto the donor interface and F-rhSP-D 70 s later. This favors the interaction of the protein with a previously-formed surfactant interfacial film at the donor compartment and allows to analyze whether, in the case of interactions with Curosurf, the interfacial spreading driving forces promote the interfacial vehiculization of F-rhSP-D. In a second scenario, F-rhSP-D was first added onto the donor interface by drop deposition and Curosurf 70 s later to evaluate whether the surfactant can somehow take the SP-D that potentially diffuses through the aqueous subphase and transport it interfacially. The interface from the recipient trough was collected after 30 min to measure the fluorescence spectra. Additionally, experiments applying OE in organic solvent (Chloroform/Methanol 2:1 v/v) were performed to avoid the formation of surface-associated structures. To do so, 20 μL of OE at 18 μg/μL (360 μg) were added by drop deposition onto the donor interface and, 10 min later for letting the organic solvents evaporate, 2.5% (9 μg; data not shown) and 5% (18 μg) of rhSP-D by mass with respect to lipids was also added on top of the donor surfactant-occupied interface by drop deposition. A Langmuir-Blodgett trough (surface area: 60–184 cm^2^; subphase volume: 350 mL) was used as recipient to transfer the interfacial film onto mica plates for AFM analysis.

### Fluorescence Spectroscopy

The vehiculization of the fluorescently-labeled F-rhSP-D by PS was detected by collecting the interface of the recipient trough and measuring the fluorescence of the covalently attached Alexa Fluor 488 (λ_excitation_ = 490 nm; λ_emission_ = 525 nm) in an Aminco Browman Series 2 spectrofluorometer. The emission spectra were measured at 25°C.

### Atomic Force Microscopy (AFM)

This technique was used to visualize the oligomers of rhSP-D that were transported by PS over the air-liquid interface. The transference of the interfacial film at the target recipient surface to mica supports was performed following two different methods: (1) by direct deposition of the mica plate on top of the interface of the recipient trough, or (2) by forming Blodgett films using a Langmuir-Blodgett trough as the recipient compartment. In the latter method, the mica plate was cleaved and submerged into the buffered subphase prior sample addition. At the end of each experiment, the mica plate was progressively raised maintaining the surface pressure constant at 20 mN/m (barrier speed: 25 cm^2^/min; dipper speed: 5 mm/min). We selected that transfer pressure in order to avoid the potential exclusion of some components and the formation of three-dimensional structures that would hinder the acquisition of images under AFM. Images were acquired using an AFM from Nanotec (Nanotec Electrónica, Madrid, Spain) with PointProbePlus tips (Nanosensors, Neuchâtel, Switzerland), or a NanoScope IIIa scanning probe microscope (Bruker, Billerica, USA) with TESP-SS tips (Bruker, Billerica, USA), in the Centro Nacional de Biotecnología (CNB, CSIC) and ICTS Centro Nacional de Microscopía Electrónica (Universidad Complutense de Madrid). Samples were imaged in tapping mode in air, at room temperature and low humidity. The images were processed using the WSxM freeware and the NanoScope Analysis software.

### Transmission Electron Microscopy

To observe the surfactant structures and confirm that PS can transport rhSP-D over air-liquid interfaces, carbon-coated cupper grids (EMS400-Cu, Gilder grids) were deposited on the interface of the recipient troughs and incubated for 30 s. Then, the grids were directly incubated for 1 min with 2% uranyl acetate (w/v) to perform negative staining. Samples were observed under a JEOL JEM-1010 transmission electron microscope (ICTS Centro Nacional de Microscopía Electrónica, Universidad Complutense de Madrid) at a magnification of 40,000x and 120,000x.

### Dynamic Light Scattering (DLS)

In order to characterize the OE samples after sonication, the hydrodynamic radius (R_H_) of aqueous suspensions in the presence or the absence of 1% rhSP-D by mass were determined using a DynaPro MS/X DLS detector equipped with a 824.7 nm-laser (Wyatt Inc). R_H_ was calculated by the Stokes-Einstein equation (Equation 1):

(1) $$\begin{array}{r} D=\frac{k_{B}\cdot T}{6\pi \eta R_{H}} \end{array}$$

where *D* is the translational diffusion coefficient, *k*_*B*_ the Boltzman constant, *T* the temperature, and η the viscosity. Water used to dilute the samples was 10 times filtered using filters of 0.22 μm (Q-Pod, Merck). Polydispersity values smaller than 15% were considered to correspond to monodisperse samples.

## Results

### Interfacial Properties and Spreading of rhSP-D Alone

**Figure 1:**
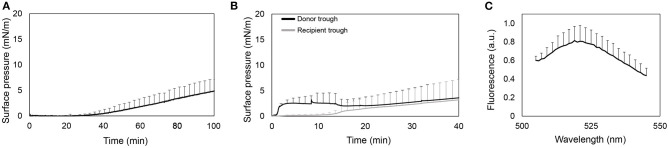
Interfacial activity of rhSP-D. **(A)** Recording of surface pressure as a function of time for the adsorption of an aliquot of 10 μL at 0.34 mg/mL (3.4 μg) of rhSP-D injected into the subphase of a Wilhelmy balance. **(B)** Adsorption and spreading isotherms of rhSP-D upon injection of an aliquot of 20 μL at 0.6 mg/mL (12 μg) of the protein at the air-liquid interface in a double-Wilhelmy balance. Surface pressure measured in the donor (black line) and recipient troughs (gray line). **(C)** Fluorescence emission spectra of the F-rhSP-D detected in the whole volume taken from the recipient trough at the end of the experiments. Data represented by the mean and standard deviations of three different replicates.

As shown in Figure 1A, the surface pressure does not increase during the first 40 min. Then, it raises to values around 5 mN/m. It indicates that the protein slowly adsorbs into the air-liquid interface, but long periods of time are required to have enough amount of protein at the interface to cause a slight increase of surface pressure.

Figure 1B shows the interfacial spreading of rhSP-D by means of changes in surface pressure during 40 min both in donor and recipient compartments. Surface pressure at donor compartment increases until stabilizing at a limited surface pressure of ~3 mN/m. Then, it slightly decreases as the surface pressure at the recipient compartment increases. The stabilization of the pressure at the donor trough and its subsequent decrease could indicate a transient adsorption of the protein into the interface and further diffusion to the recipient trough. However, once the surface pressure at the recipient equals the pressure at the donor compartment, the latter increases as well, indicating a continuous adsorption of rhSP-D at the donor interface until the interface stabilizes. To confirm the presence of the protein in the recipient trough, interfacial films were transferred to carbon-coated cupper grids and observed by TEM (Supplementary Figure 2), but no traces of SP-D were observed. Therefore, we also performed the vehiculization assays using the fluorescent derivative of rhSP-D. In this case, the smallest recipient trough was used to collect the whole volume (1.8 mL) and also measure the F-rhSP-D that might diffuse and dilute away from the interface into the subphase. As observed in Figure 1C, fluorescence was detected in the recipient compartment, suggesting that rhSP-D may actually cross the bridge alone from the donor to the recipient trough. However, the fluorescent signal was very low and maximal sensitivity in the fluorometer was required to detect it.

### Interfacial Delivery of rhSP-D in the Presence of Pulmonary Surfactant

Once analyzed the adsorption and spreading properties of the rhSP-D alone confirming low interfacial adsorption and spreading capabilities, the next step was to analyze how the presence of PS influences the interface-assisted vehiculization process. To do so, different strategies were followed including a PS/rhSP-D combined formulation and the addition of PS and rhSP-D separately.

#### Interfacial Vehiculization of a Combined PS/rhSP-D Formulation

**Figure 2:**
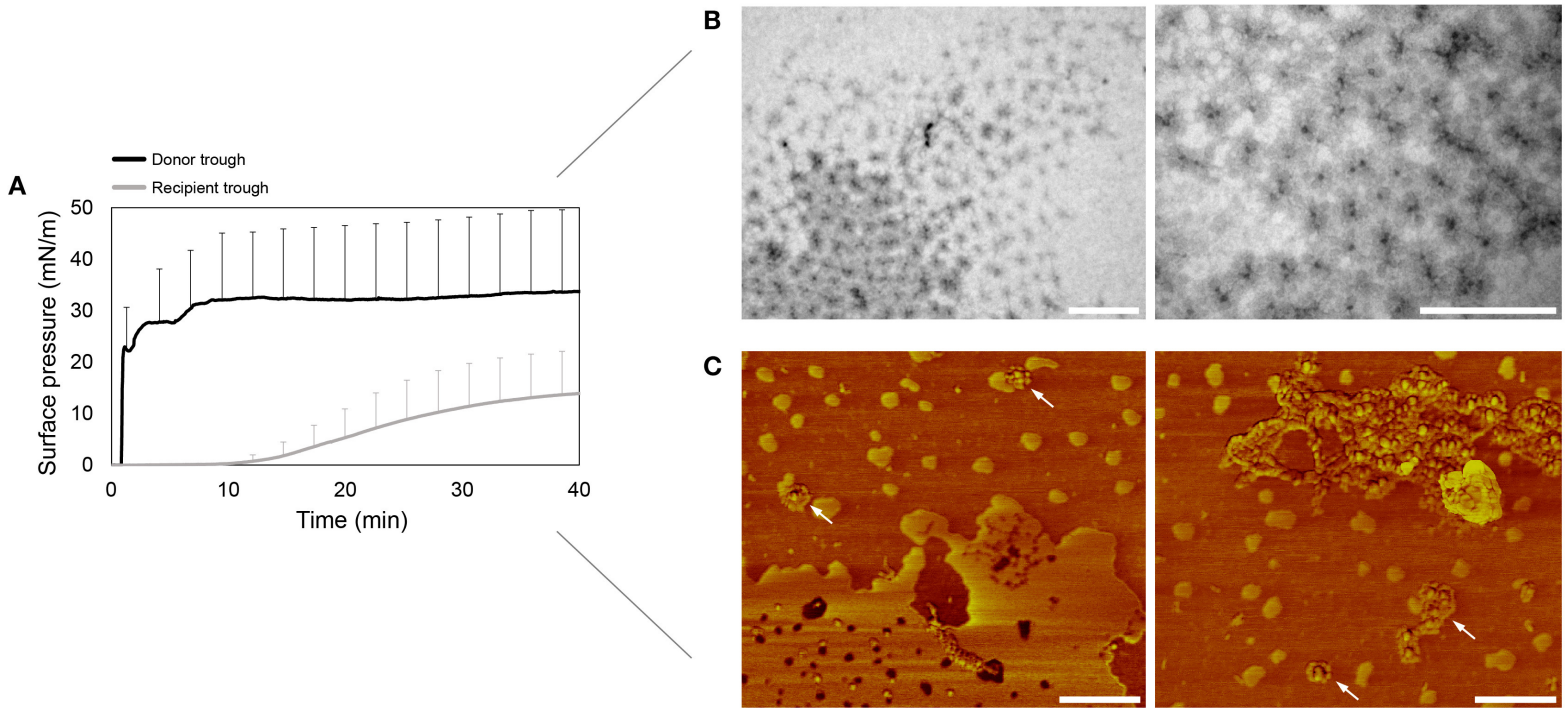
Pulmonary surfactant vehiculization of rhSP-D over the air-liquid interface. **(A)** Adsorption and spreading isotherm of a suspension of the organic extract from native surfactant (OE) reconstituted and mixed with rhSP-D at 1% protein/lipid (w/w) ratio. An aqueous aliquot of 15 μL (50 mg/mL; 750 μg) of the material were deposited dropwise at the donor interface in the double-Wilhelmy balance and changes in surface pressure were measured in both troughs. Mean and standard deviations were obtained from three replicates. **(B)** Transmission electron microscopy (TEM) micrographs of the rhSP-D fuzzy balls detected at the recipient air-liquid interface upon surfactant-promoted interfacial vehiculization. **(C)** rhSP-D vehiculized by surfactant detected at the recipient air-liquid interface by atomic force microscopy (AFM) phase images. Some examples of fuzzy balls are pointed with white arrows. Scale bar: 400 nm.

Figure 2A shows that right after addition of OE/rhSP-D mixture, the surface pressure at the donor compartment increases sharply above 30 mN/m, indicating a proper interfacial adsorption of the formulation. After 10 min, the surface pressure in the recipient trough starts increasing as well, though this increase seems to be lower than the one observed in the donor compartment. This, together with the high error bars could indicate that rhSP-D could somehow affect or modulate the adsorption and spreading capabilities of pulmonary surfactant, something that needs further exploration to elucidate the relevant factors involved in this potential effect. In spite of the donor-to-recipient diffusion of OE/rhSP-D, the surface pressure at the donor trough always remained stable, indicating a rapid and continuous adsorption and spreading of new material from the surface-associated reservoirs at the donor compartment.

To confirm the potential of OE to transport rhSP-D over the air-liquid interface, the material placed at the recipient interface was transferred onto carbon-coated cupper grids and mica plates for TEM and AFM visualization, respectively. The micrographs obtained by TEM (Figure 2B) shows accumulation at the interface of fuzzy-ball-like structures, recognizable by the higher electron density of the central N-terminal collagenous stem. These structures are similar to the ones observed somewhere else (Holmskov, 2000). The AFM phase images (Figure 2C) demonstrated the presence of rhSP-D fuzzy balls at the air-liquid interface, appearing both grouped and isolated.

#### Understanding the Mechanisms Behind the Interaction and Interfacial Spreading of PS and rhSP-D

**Figure 3:**
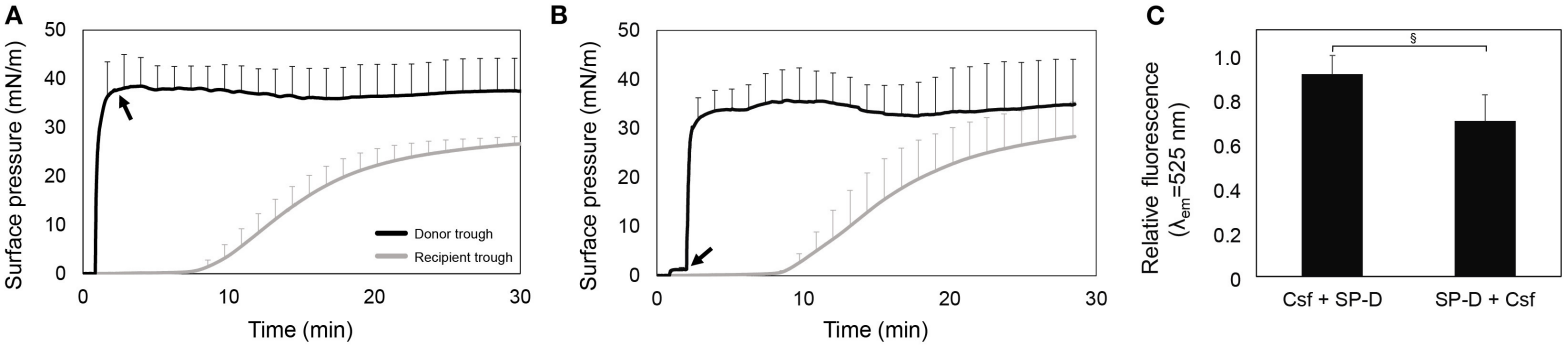
rhSP-D conjugated with Alexa Fluor 488 transported over the interface by the association with Curosurf (Csf). **(A)** Adsorption and spreading isotherm obtained from the interfacial injection of 50 μL (80 mg/mL; 4 mg) of Curosurf and, 70 s after, 15 μL (1 mg/mL; 15 μg) of F-rhSP-D (black arrow), measured in the double-Langmuir balance. **(B)** Pressure-time isotherm upon injection of, first, F-rhSP-D and, 70 s after, Curosurf (black arrow), in a double-Langmuir balance. **(C)** Relative fluorescence emission at λ_em_ = 525 nm of the material collected from the recipient interface by aspiration at the end of each experiment. Data represent mean and standard deviation calculated from three different experiments. Pair *t*-test: (§) *p* = 0.078.

Figures 3A,B show ∏-time isotherms adding first Curosurf or F-rhSP-D, respectively. In both scenarios, Curosurf reaches the equilibrium surface pressure (around 40 mN/m) in the donor compartment right after injection, and subsequently the surface pressure in the recipient trough also increased. The injection of F-rhSP-D after 70 s in the presence of the preformed surfactant film does not induce further changes in surface pressure either in the donor neither in the recipient. This indicates that the protein does not affect the interfacial and spreading properties of Curosurf. After 30 min, the recipient interface was collected to measure the fluorescence of F-rhSP-D. As shown in Figure 3C, F-rhSP-D was detected at the recipient trough in both scenarios. Although no statistically significant differences were observed (*p* = 0.078), the injection of Curosurf prior to the protein seems to show a tendency to enhance the vehiculization, which could indicate a more extensive interaction with rhSP-D at the air-liquid interface when surfactant structures are already adsorbed at the interface.

**Figure 4:**
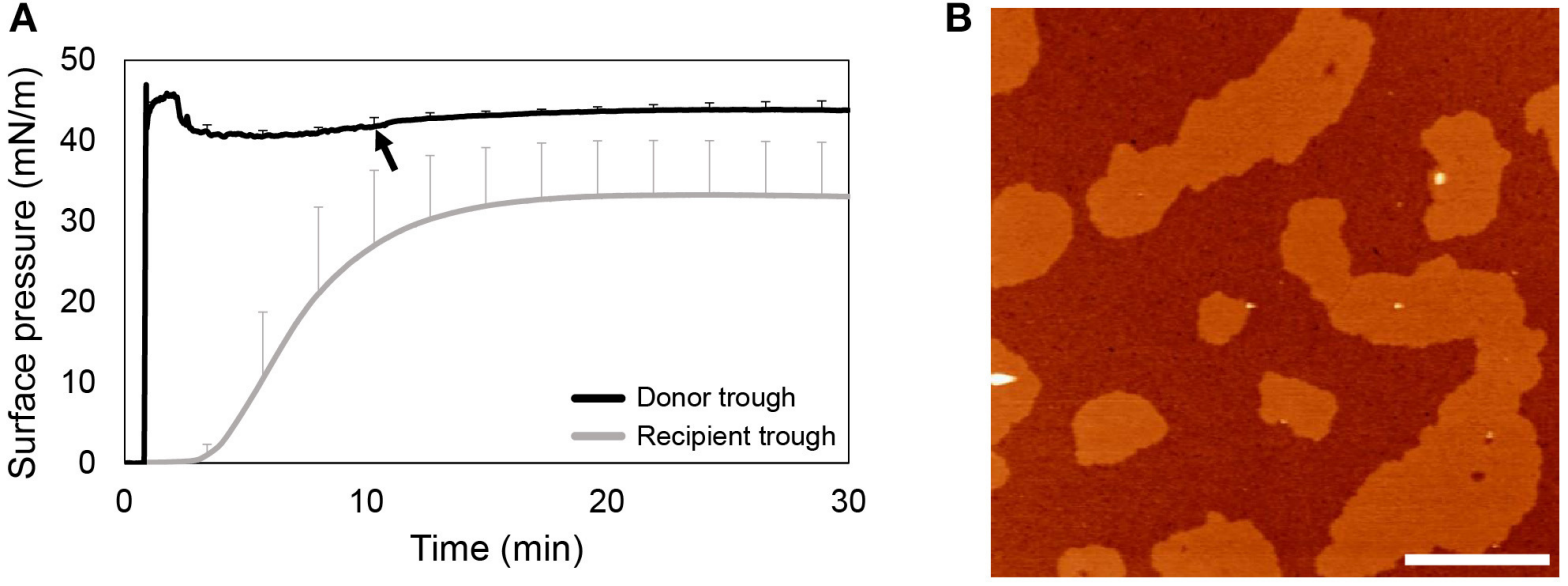
rhSP-D association to the interface in the absence of surfactant-associated reservoirs. **(A)** Spreading isotherm of OE in organic solvent in a double-Langmuir balance and **(B)** AFM height image obtained from the transference of the recipient interfacial film after the spreading of 360 μg of OE in organic solvent and the injection of 5% w/w rhSP-D 10 min after (black arrow in **A**). Three replicates were performed to obtain mean and standard deviation data.

To avoid formation of surface-associated reservoirs and to have both donor and recipient interfaces saturated with surfactant and stable prior to the addition of rhSP-D, OE in organic solvent was firstly applied onto the donor air-liquid interface. Figure 4A shows that OE rapidly spreads over the interface, reaching and stabilizing at the equilibrium surface pressure. Then, to allow organic solvent to evaporate, rhSP-D was applied at the donor interface 10 min later. At the end of the experiment, the recipient interface was transferred onto a mica plate for detecting the presence of rhSP-D by AFM analysis. As observed in the images shown in Figure 4B, rhSP-D was not detectable at the recipient interface.

#### Differential Vehiculization of Oligomeric Forms of rhSP-D by Pulmonary Surfactant

In an attempt to elucidate whether the different oligomers are transported differently and whether surfactant structure could influence this process, vehiculization of the OE/rhSP-D combination was assessed with sonicated and non-sonicated OE suspensions. The sonication process favors the formation of smaller surfactant vesiculated structures with higher curvature (García-Fojeda et al., 2019), which has been proposed to promote interaction of amphiphilic proteins. As observed in Supplementary Figure 3, sonication induced fragmentation of OE vesicles observable by means of more monodispersed population of smaller vesicles. The presence of rhSP-D caused a shift in the peak to larger sizes in both sonicated and non-sonicated surfactant, indicating an interaction of rhSP-D with surfactant membranes.

**Figure 5:**
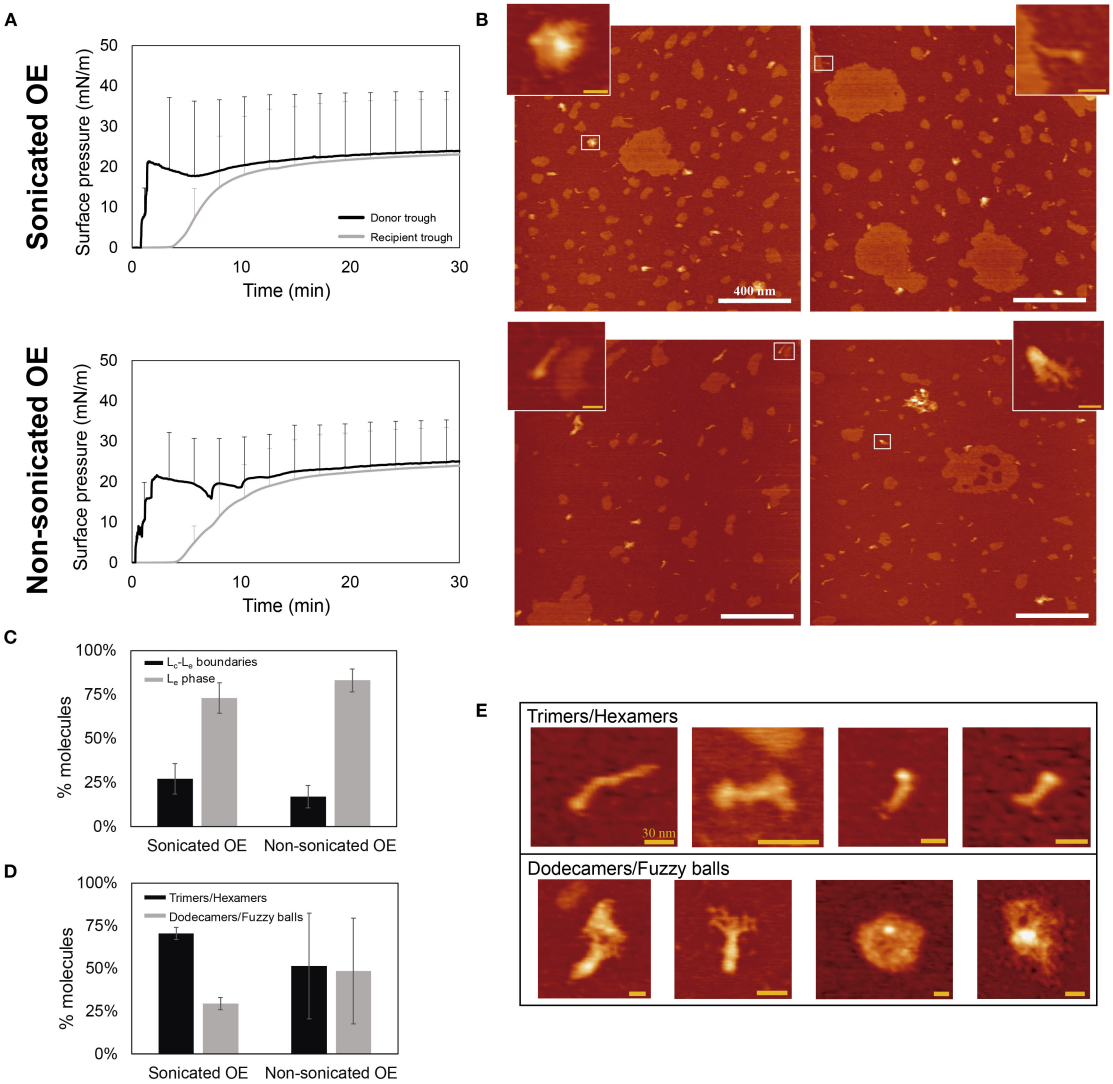
Analysis of rhSP-D oligomers transported over the air-liquid interface associated to surfactant complexes. An aqueous aliquot of OE/rhSP-D at 50 mg/mL (750 μg) and 1% rhSP-D by mass (7.5 μg) was applied at the donor interface. **(A)** Adsorption and spreading isotherms performed in the double-Langmuir balance. **(B)** AFM height images taken after transference onto mica surface of the recipient interfacial film. Data obtained by the vehiculization of sonicated (top) or non-sonicated (bottom) OE before mixing with 1% w/w rhSP-D. Scale bar: 400 nm. Zoom regions show examples of rhSP-D oligomers corresponding to the areas highlighted with white rectangles. Scale bar: 30 nm. **(C)** Percentage of rhSP-D oligomers found at the L_c_-L_e_ boundaries (black) or distributed into the L_e_ phase (gray) upon vehiculization by sonicated or non-sonicated surfactant. **(D)** Quantitative distribution of smaller and larger rhSP-D oligomers observed in surfactant films, upon association of the protein with sonicated or non-sonicated surfactant structures. **(E)** Representative AFM images of the different SP-D oligomers grouped into trimers/hexamers and dodecamers/fuzzy balls. Scale bar: 30 nm.

Figure 5A compares the ∏-time isotherms of both donor and recipient compartments upon application of sonicated or non-sonicated samples. After 30 min, the interfacial film at the recipient trough was transferred onto a mica plate at a constant surface pressure of 20 mN/m to avoid the formation of multilayered structures and the exclusion of material from the interface once higher pressures are reached. The surfactant vehiculization of rhSP-D by both approaches was demonstrated by observing the presence of rhSP-D oligomers under the AFM (see Figure 5B). Coexistence of liquid-condensed (L_c_) and liquid-expanded (L_e_) lipid phases are differentiable in Figure 5B, where L_c_ domains exhibit round-shaped areas with an average difference in height of 5.5 ± 0.89 Å (mean ± SD) surrounded by more extended L_e_ phases (see Supplementary Figure 4A), consistent with previous observations (Yuan and Johnston, 2002; Blanco et al., 2012). The percentage of area that occupied big (>200 nm) or small (<200 nm) L_c_ domains was also analyzed, but no differences were observed between the films formed by sonicated or non-sonicated samples (Supplementary Figure 4B). SP-D molecules are predominately distributed associated with the L_e_ phase compared with the fraction of the protein seen associated with L_c_-L_e_ boundaries (Figure 5C). Interestingly, the protein seems to present a closed configuration of their collagenic arms, and seems to be at least partly buried into the lipid film, observed as protein molecules with similar height but shorter in length than previously described (Arroyo et al., 2018) (Supplementary Figure 4C). This particular configuration makes difficult to identify the number of trimers taking part of each oligomer. To assess whether smaller or larger oligomeric forms were preferentially transported by interfacial films assembled from smaller or larger surfactant vesicles, we quantified the oligomers including trimers or hexamers on one group and higher ordered dodecamers and fuzzy balls on the other (see Figures 5D,E). In both cases, when using sonicated or non-sonicated surfactant suspensions, an apparently larger number of trimers/hexamers were transported from the donor to the recipient trough in comparison with the proportion of dodecamers and fuzzy balls vehiculized and with the proportion of smaller and larger oligomers in this preparation when examined on plain mica (roughly 50% of each). No significant differences were observed when comparing sonicated and non-sonicated samples, although the intrinsic variability of the few replicas examined prevents a clear conclusion at this stage. The proportion of SP-D trimers and hexamers observed as associated with the interfacial film seems to be higher when smaller surfactant vesicles were accessible to the protein, possibly indicating a trend of the smaller oligomers to interact better with highly curved membranes.

## Discussion

Pulmonary surfactant protein SP-D plays essential roles in alveolar immunity and surfactant metabolism (Clark and Reid, 2003). However, the current clinical surfactants used for surfactant replacement therapy (SRT) to treat infant respiratory distress syndrome (RDS), a common cause of morbidity and mortality in preterm neonates characterized by pulmonary immaturity and lack of PS, lack the hydrophilic collectins SP-A and SP-D (Johansson and Curstedt, 2019; Hentschel et al., 2020). Therefore, in this study we have investigated the possibility that protein SP-D could interact with interfacial surfactant films and, through the interface, diffuse over the whole respiratory surface, which could facilitate its function to encounter, interact and label for clearance potential harmful entities impinging the surfactant film, the first barrier exposed to the outer environment in the lungs. In the study, we have used a recombinant form of human SP-D. Our experiments are therefore also useful to show how the combination of the protein with PS could be a useful strategy to facilitate an efficient delivery of the protein through the airways as a therapeutic option, using PS as a shuttle. The use of PS as a drug delivery system to carry and distribute different therapeutic molecules over the respiratory surfaces have been studied in the recent years both *in vitro* and *in vivo* (Van't Veen et al., 1996; Hidalgo et al., 2017, 2020; Baer et al., 2018). The combination of exogenous PS with rhSP-D could have the potential to serve as a preventive or therapeutic approach to treat inflammatory responses and lung diseases in preterm infants such as RDS or bronchopulmonary dysplasia (BPD) (Ikegami et al., 2007; Sato et al., 2010).

The research about SP-D has been focused around its immune roles and anti-inflammatory properties (Crouch et al., 1995; Cai et al., 1999; Liu et al., 2005; Ikegami et al., 2006; Cohen et al., 2020), but little is known about its interfacial properties and its potential combination with PS to complement anti-inflammatory actions, or to define novel therapeutic approaches through the airways. In this work, we report a low interfacial adsorption and spreading properties of rhSP-D by itself on clean air-water interfaces. However, in the presence of pulmonary surfactant, either delivered as a PS/rhSP-D combined formulation or co-administered one right after the other, rhSP-D efficiently traveled associated to air-liquid interfaces. Although the combination of rhSP-D with PS seems to slightly affect the interfacial performance of PS revealed by lower surfaces pressures reached at the recipient compartment and larger experimental variability, it is clear that the mixed formulation favors the interaction and permanence of the protein at the interface and, consequently, its spreading over it (Figure 2). When rhSP-D was applied with the donor interface already occupied by PS, it was also detected in the recipient compartment, indicating that the protein is able to interact with pre-existing surfactant films at the interface and used them as a sort of shuttle to rapidly spread long distances via the interface. Similarly, the fact that adding PS right after rhSP-D also promoted the interfacial vehiculization of the protein, in contrast to the poor interfacial spreading of rhSP-D alone (Figures 1, 3), suggests that the protein can shift from a free form in the aqueous bulk phase to a lipid-associated state that is competent to diffuse over the interface. We propose that SP-D/lipid complexes, or alternatively, the interaction of SP-D with any of the hydrophobic surfactant proteins present in the film, converts SP-D into a form that is stably associated with the interface and facilitates its “surfing” capabilities. The injection of rhSP-D on top of a pre-formed surfactant film that had reached surface pressure values of around 15 mN/m, produced an instantaneous and visible increase in surface pressure, confirming the rapid adsorption of the protein into the interface and its insertion into the surfactant film (data not shown). These observations are consistent with the effect on the initial surface pressure as a consequence of SP-D adsorption that was described by Taneva et al. (1997). At surface pressures above ~30 mN/m, SP-D, as occurring with other hydrophilic proteins, cannot penetrate into the lipid films. Thus, rhSP-D may somehow attach to either PS at the interface or the PS reservoirs at the subphase, most likely through the interaction of its CRD with PS phospholipids (Ogasawara et al., 1992; Persson et al., 1992), and leverage the interfacial spreading forces even without their previous combination. This opens the possibility to deliver rhSP-D as a mixed formulation together with PS or administered in a close time window but separately one after the other, without the necessity to develop *de-novo* PS/rhSP-D combined formulations, which could reduce time and costs associated with the design and implementation of clinical trials *ad hoc*.

Nonetheless, when rhSP-D was applied with both donor and recipient interfaces completely saturated with PS to emulate the physiological conditions, the protein was not detected in the recipient compartment (Figure 4). This could indicate that SP-D could be able to interact and spread mainly in physiological contexts where surfactant has been depleted from the interface for some reason. However, the absence of breathing-like interfacial compression/expansion dynamics in the current experiments could limit the behavior of SP-D compared with the potential action of the protein *in vivo*. We have recently demonstrated that breathing dynamics could be essential to understand the interfacial behavior of surfactant, particularly with respect to potential interface-assisted spreading capabilities and release processes (Hidalgo et al., 2020), as a consequence of surface tension-driven interfacial flows and the potential progressive exclusion of material from the interface (Borgas and Grotberg, 1988; Pastrana-Rios et al., 1994; Grotberg and Gaver III, 1996; Halpern et al., 1998; Keating et al., 2012; Hidalgo et al., 2020). These effects could be important to promote the spreading of new material coming from upstream reservoirs and better distribute the therapeutics over the respiratory surface (Hidalgo et al., 2020). Thus, further experiments are needed to explore the interfacial delivery of rhSP-D in saturated interfaces subjected to breathing-like dynamic conditions.

The structures of SP-D identified in the images taken by TEM and AFM (Figures 2, 5) are consistent with those obtained in previous studies (Holmskov, 2000; Arroyo et al., 2018), though the association/vehiculization with PS seem to modulate their conformation slightly. All the oligomers analyzed presented a closed conformation, with the collagen domains and the CRD heads less defined. This is likely a consequence of their association with phospholipid surfaces, as it occurs with other hydrophilic proteins (Maget-Dana and Ptak, 1995), something that should be investigated in more detail. We also found a differential vehiculization of the different rhSP-D oligomers over the air-liquid interface. Trimers and hexamers are apparently better transported associated to pulmonary surfactant than dodecamers and fuzzy balls (Figure 5). This can be related with their smaller size and a facilitated diffusion associated with the interfacial film. This effect could have some consequences on the role of SP-D in PS homeostasis. SP-D seems to be involved in the regulation of surfactant lipid pool sizes, contributing somehow to the transformation of surfactant large aggregates into small aggregates, preferentially taken up by alveolar type II pneumocytes but not macrophages (Horowitz et al., 1997; Ikegami et al., 2000). Still, when higher order oligomers-enriched batches, the most active oligomers in bacterial aggregation (Arroyo et al., 2020), were used (Figure 2), PS was also able to transport them efficiently.

Although our data suggests that PS improves the travel of SP-D across air-liquid interfaces, the Wilhelmy balance results may not be identical to the properties of the alveolar air-liquid interface. In addition, it is uncertain how much PS is needed to facilitate SP-D movement. It is possible that PS levels may be sufficient to achieve maximum SP-D distribution even in surfactant depleted conditions such as RDS.

The above limitations notwithstanding, altogether, this work points out the potential synergistic effect that PS/rhSP-D formulations could have to empower surfactant replacement therapy (SRT) to treat infants with RDS or BPD. It could also offer new possibilities to use SRT in acute lung injuries such as acute respiratory distress syndrome (ARDS) or lung infections in both children and adults. The administration of exogenous surfactants either animal-derived (Kesecioglu et al., 2009) or synthetic (Spragg et al., 2004) has failed so far for treating ARDS, possibly, at least in part, due to the presence in the airways of surfactant inhibitors such as serum components or phospholipases (Autilio et al., 2020) derived from severe inflammation processes and the damage of alveolar epithelium. The incorporation of recombinant forms of human SP-D could contribute to mitigate the inflammation process at the distal airways and enhance the efficacy of SRT. Interestingly, SP-D has also demonstrated different anti-infective activities including antifungal actions (Madan et al., 2001; Ordonez et al., 2019), abilities to recognize and promote virus and bacterial killing and clearance (Crouch, 2000; Hillaire et al., 2013) and specifically binding to the highly glycosylated S-protein of coronavirus inhibiting their replication (Leth-Larsen et al., 2007). Thus, an efficient administration of SP-D could also be beneficial for the treatment of diseases associated with lung infection such as the current COVID-19 pandemic caused by the SARS-CoV-2 virus. The administration of SP-D combined with PS to patients suffering from severe ARDS could help to mitigate lung inflammation and counteract the secondary bacterial and viral infection. In summary, the optimization of PS/rhSP-D formulations could be interesting to empower the current clinical surfactants increasing their potential to replace the lack or damaged endogenous surfactant, to open damaged and poorly-aerated areas in the lungs and to act as a carrier distributing rhSP-D over the respiratory surface. A similar principle could be explored to optimize surfactant-promoted vehiculization of other therapeutic proteins along the interface, including versions of the proteins that could be modified to facilitate their association with surfactant and a efficient interface-driven vehiculization through the airways.

## Data Availability Statement

The original contributions presented in the study are included in the article/Supplementary Materials, further inquiries can be directed to the corresponding author.

## Author Contributions

CG-M and AH designed the study, acquired data by performing most of laboratory experiments, interpreted data, and drafted the manuscript. RA acquired data by performing some experiments. ME and AC supervised some data analyses. JP-G conceptualized the study, supervised the whole work, and interpreted all the data. All authors critically revised the paper for important intellectual content and finally approved the paper in the present form. All authors agreed to be accountable for all aspects of the work in ensuring that questions related to the accuracy or integrity of any part of the work were appropriately investigated and resolved.

## Conflict of Interest

RA was paid as part-time consultant by Airway Therapeutics Inc. JP-G has received research grants from Chiesi Farmaceutici spa and Airway Therapeutics. The remaining authors declare that the research was conducted in the absence of any commercial or financial relationships that could be construed as a potential conflict of interest.
