# Supplementary material for: Pulmonary Surfactant and Drug Delivery: An Interface-Assisted Carrier to Deliver Surfactant Protein SP-D Into the Airways
Source: Front Bioeng Biotechnol. 2021 Jan 18;8:613276. doi: 10.3389/fbioe.2020.613276 (PMC7853302; doi:10.3389/fbioe.2020.613276)
Supplement: Supplementary file 1 [file Presentation_1.pdf]

Supplementary Material

Supplementary Figures

Supplementary Figure S1

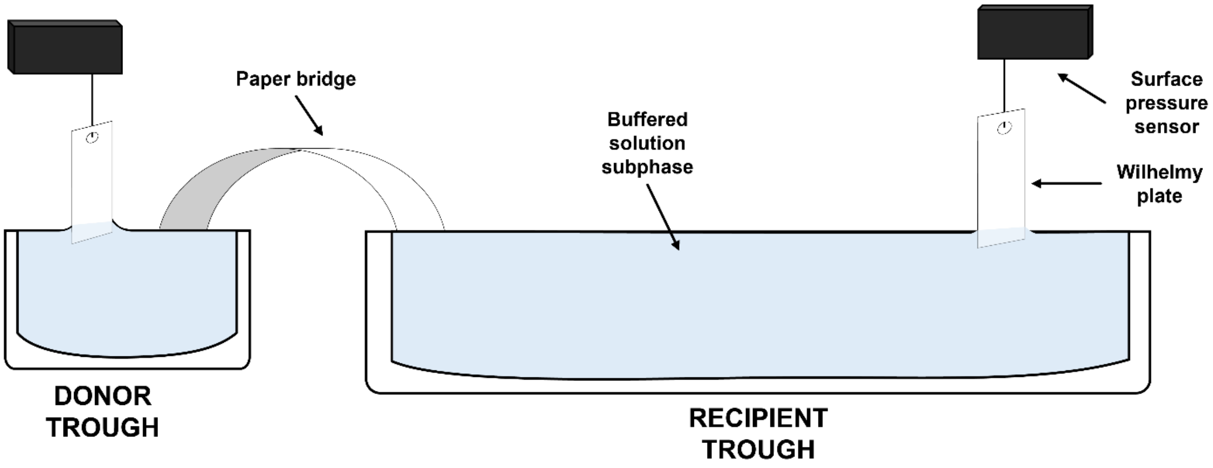

Figure S1. Schematic representation of the vehiculization setup consisting in two different aqueous compartments connected by an interfacial bridge. One of the compartments acts as a donor where the sample is applied, somehow emulating the upper airways. Surface active materials able to spread over air-liquid interfaces travel along the interfacial bridge (hydrated filter paper), which models the conductive airways, reaching the recipient compartment that mimics the distal airways. The interfacial spreading is continuously monitored by recording the surface pressure values in both troughs with two pressure sensors, at a controlled temperature. To determine the vehiculization properties of PS, the presence of the transported molecules at the recipient compartment can be analyzed.

Supplementary Figure S2

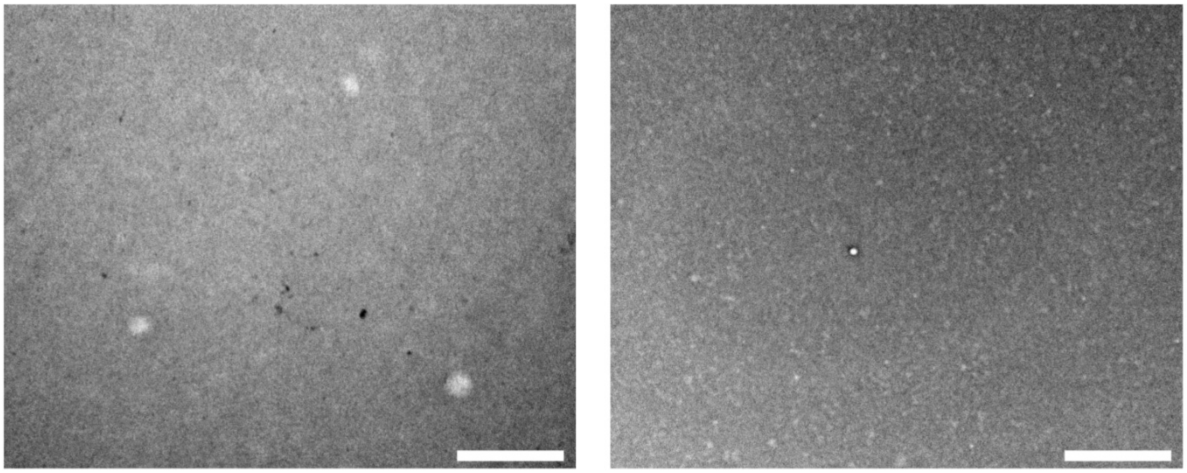

Figure S2. Spreading of rhSP-D over the interface of the double-Wilhelmy balance. TEM images of the donor (left) and recipient (right) interface after the injection of an aliquot of 20 μL at 0.6 mg/mL of rhSP-D at the donor trough. The scale bar represents 1 μm.

Supplementary Figure S3

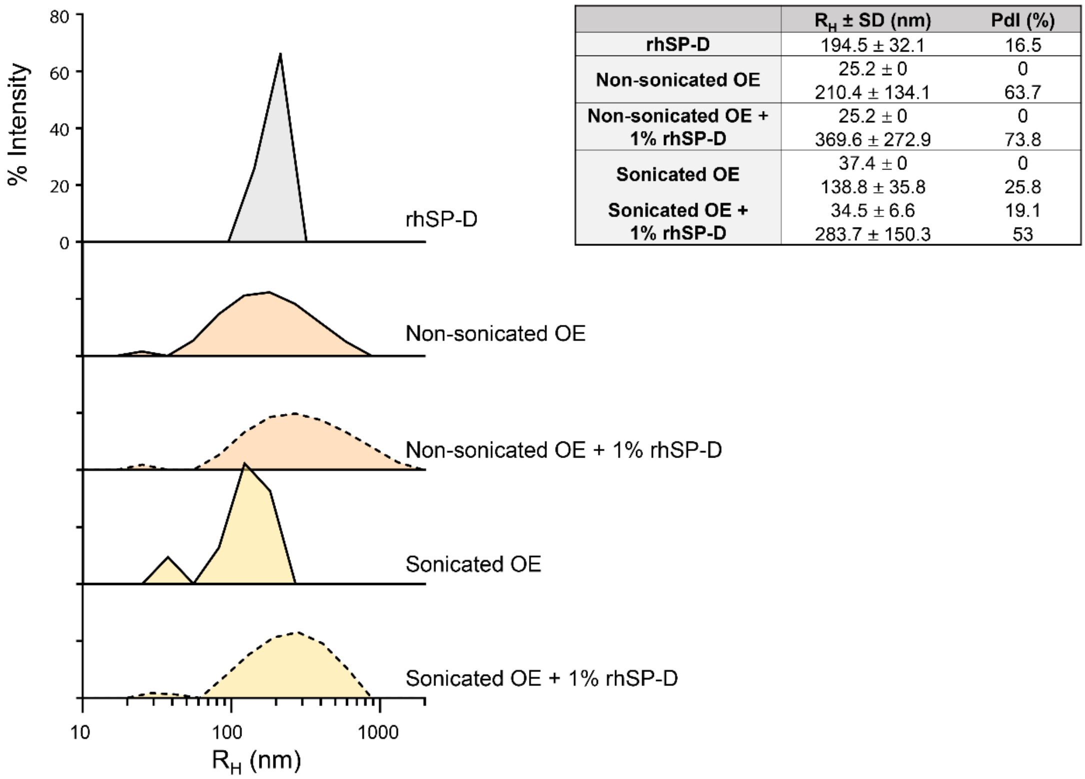

	$R_H \pm$ SD (nm)	PdI (%)
**rhSP-D**	194.5 ± 32.1	16.5
**Non-sonicated OE**	25.2 ± 0	0
	210.4 ± 134.1	63.7
**Non-sonicated OE + 1% rhSP-D**	25.2 ± 0	0
	369.6 ± 272.9	73.8
**Sonicated OE**	37.4 ± 0	0
	138.8 ± 35.8	25.8
**Sonicated OE + 1% rhSP-D**	34.5 ± 6.6	19.1
	283.7 ± 150.3	53

Figure S3. Hydrodynamic radius (R_H) distribution determined by dynamic light scattering (DLS). The aqueous suspension of OE was sonicated during 2 min (burst for 0.6 s, and 0.4 s between bursts) at 65% amplitude for 7 cycles in a UP 200S sonifier, with a 2 mm microtip. The material was then incubated with 1% w/w rhSP-D at 37 ºC for 30 min. Data table shows the mean R_H ± standard deviation (SD) and poly-dispersity index (PdI) of sonicated and non-sonicated OE suspension before and after mixing with 1% w/w rhSP-D.

Supplementary Figure S4

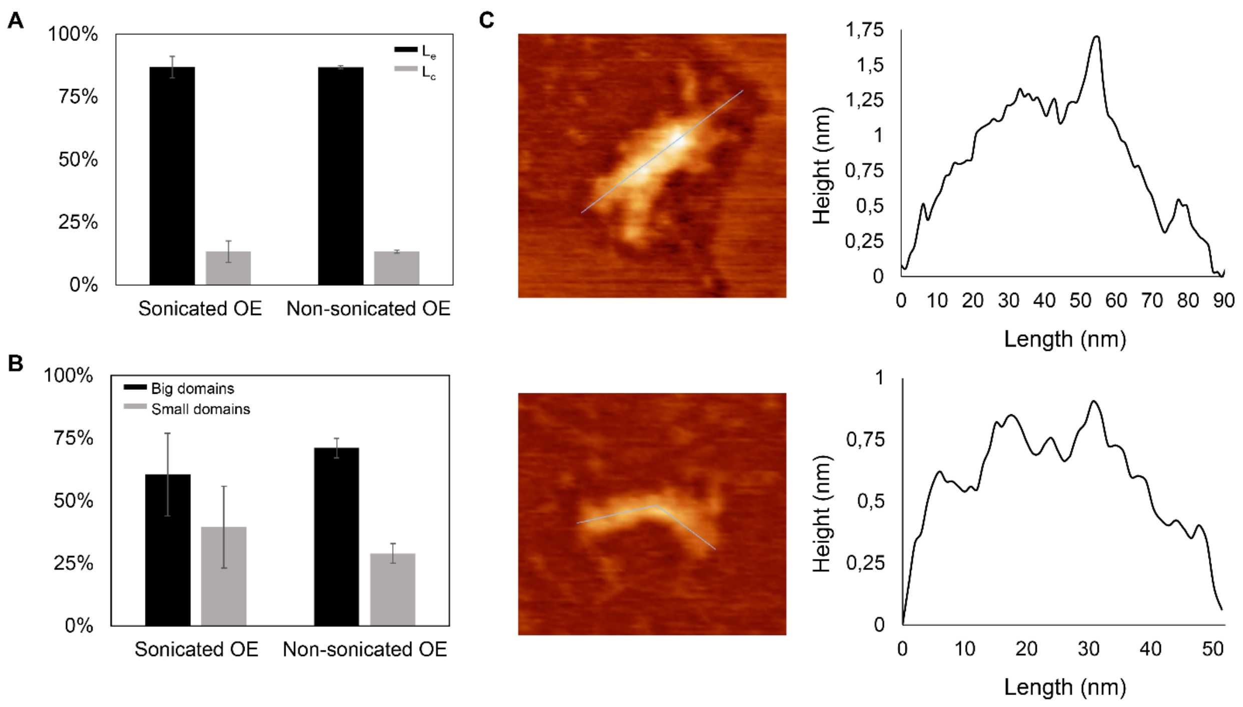

Figure S4. Analysis of AFM images taken from the transfer of material at the recipient interface upon vehiculization of rhSP-D associated with sonicated or non-sonicated OE. (A) Mean percentage of total area covered by L_e (black) and L_c (grey) phases in both conditions. The total area occupied by all L_c domains was measured and subtracted from the total area of each image to obtain the area occupied by L_e phase. (B) Mean percentage of area occupied by big (> 200 nm diameter, black) or small (< 200 nm diameter, grey) L_c domains from the total L_c area analyzed in images taken from the recipient surface films formed upon spreading of sonicated or non-sonicated OE suspensions. The area of every L_c domain was measured and accumulated to calculate the percentage from the total L_c area covered by either big or small domains for each condition. (C) Example of height profiles taken along the grey line drawn in illustrative images of a dodecamer/fuzzy ball (up) and a trimer/hexamer (bottom) oligomer of rhSP-D.
